# Multivariate characterization of the nutritional diversity of traditional Ecuadorian foods

**DOI:** 10.3389/fnut.2026.1913247

**Published:** 2026-08-06

**Authors:** Orlando Meneses Quelal

**Affiliations:** Carrera de Alimentos, Facultad de Industrias Agropecuarias y Ciencias Ambientales, Universidad Politécnica Estatal del Carchi (UPEC), Tulcán, Ecuador

**Keywords:** Ecuador, food composition, multivariate analysis, nutritional diversity, principal component analysis, traditional foods

## Abstract

The availability of food composition databases is a fundamental tool for nutritional research, public health, and food policy design. However, the multivariate structure of the nutritional diversity of traditional Ecuadorian foods remains understudied. The objective of this research was to characterize the nutritional composition of traditional Ecuadorian foods using descriptive, inferential, and multivariate analyses. The Food Composition Table of Cuenca, Ecuador, comprising 129 foods distributed across ten food groups, was used. Because this database was compiled from foods sampled primarily in the southern Andean region of Ecuador, the findings characterize the nutritional structure of this regional database rather than Ecuador as a whole. Ten nutritional variables related to energy, macronutrients, and minerals were analyzed. Differences between groups were evaluated using one-way ANOVA, while relationships between nutrients were studied using Pearson correlations. Additionally, principal component analysis (PCA) and hierarchical cluster analysis were applied to identify nutritional patterns. The results showed significant differences between food groups for energy (*F* = 23.71; η^2^ = 0.614), protein (*F* = 20.38; η^2^ = 0.641), carbohydrates (*F* = 18.61; η^2^ = 0.560), fat (*F* = 8.97; η^2^ = 0.491), calcium (*F* = 3.00; η^2^ = 0.178), and zinc (*F* = 11.15; η^2^ = 0.446) (*p* < 0.05). The strongest correlation was observed between energy and water (*r* = −0.972), followed by energy and carbohydrates (*r* = 0.783). The PCA showed that the first two components explained 49.6% of the total variability and revealed two main gradients: energy density and protein-mineral profile. Cluster analysis identified four distinct nutritional profiles. The results demonstrate that the nutritional diversity of Ecuadorian foods is organized around well-defined compositional patterns, providing a scientific basis for future applications in nutrition, epidemiology, and sustainable food systems.

## 1 Introduction

The chemical composition of food is a fundamental pillar of human nutrition, nutritional epidemiology, food policy formulation, and the development of food security strategies (1). The availability of reliable information on energy, macronutrient, and micronutrient content allows for the assessment of the nutritional quality of diets, the estimation of nutrient adequacy in different population groups, and the design of evidence-based interventions (2). For this reason, food composition databases represent an essential scientific infrastructure for nutritional research and public health worldwide.

Over the past few decades, international organizations such as the Food and Agriculture Organization of the United Nations (FAO), INFOODS, and LATINFOODS have promoted the development and harmonization of food composition databases to improve the quality, comparability, and accessibility of nutritional information available to researchers, healthcare professionals, and policymakers (3). These initiatives have consolidated thousands of nutritional records for foods from different geographic regions, facilitating comparative studies on dietary patterns, food biodiversity, and the nutritional transition.

The usefulness of these databases goes beyond simply describing individual nutrients. Foods constitute complex biological matrices in which energy, proteins, fats, carbohydrates, minerals, and other bioactive compounds interact simultaneously, generating specific nutritional profiles that are difficult to understand through isolated univariate analyses (4). Consequently, in recent years there has been an increased use of multivariate statistical tools to explore patterns of association between nutrients, identify nutritional profiles, and classify foods according to their overall chemical composition (5).

Among these tools, principal component analysis (PCA) has proven particularly useful for reducing the dimensionality of large nutritional datasets and revealing underlying compositional gradients that are not evident through conventional descriptive analyses (6). Complementarily, cluster analyses allow for the grouping of foods with similar nutritional characteristics, facilitating the identification of dietary patterns and functional food profiles (7). The integration of correlation analysis, PCA, and hierarchical clustering has been widely applied in food composition studies conducted in Europe, Asia, and Latin America, providing a more comprehensive view of the nutritional organization of food systems (8).

In Ecuador, biological, climatic, and cultural diversity has historically favored the availability of a wide variety of plant and animal-based foods (9). The coexistence of Andean, Amazonian, coastal, and island ecosystems has contributed to the development of highly diverse food systems, characterized by the presence of traditional grains, tubers, legumes, tropical fruits, livestock products, and a wide range of regional dishes. This diversity constitutes a food heritage of considerable nutritional and cultural significance (10, 11).

Despite this rich food supply, studies focused on the multivariate characterization of the nutritional composition of Ecuadorian foods remain limited (12). Most available research has focused on the analytical determination of specific nutrients or the compilation of food composition tables, while analyses aimed at understanding the overall structure of nutritional variability have received less attention (13). Consequently, there is still limited knowledge about the main nutritional gradients that organize traditional Ecuadorian foods and how different food groups contribute to the observed nutritional diversity.

The Cuenca Food Composition Table is currently one of the most comprehensive sources of nutritional information available for traditional Ecuadorian foods. This database compiles analytical information obtained through standardized methodologies for a wide variety of foods consumed in the Andean region of the country, providing a valuable opportunity to explore nutritional patterns using advanced statistical approaches (14). However, to date, no comprehensive analyses combining descriptive statistics, inferential analysis, and multivariate techniques have been conducted to characterize the underlying nutritional structure of this database.

In this context, the objective of this study was to characterize the nutritional composition of traditional Ecuadorian foods using descriptive, inferential, and multivariate analyses. Specifically, nutritional differences between food groups were evaluated using analysis of variance, relationships between nutrients were analyzed using Pearson correlations, compositional gradients were identified using principal component analysis, and nutritional profiles of foods were determined using hierarchical cluster analysis. This approach allowed for an integrated view of the nutritional diversity present in traditional Ecuadorian foods and provided useful evidence for future applications in nutrition, public health, and sustainable food systems.

## 2 Materials and methods

### 2.1 Data source and database construction

This study was conducted using the Food Composition Table of Cuenca, Ecuador, developed by the Food, Nutrition, and Health Research Group at the University of Cuenca (15). The original database contains nutritional information for 129 locally produced foods and traditional dishes primarily representative of the southern Andean region of Ecuador. Although the database includes many foods traditionally consumed in other regions of the country, its analytical sampling was primarily conducted in the southern Andean region. The data were generated progressively between 2011 and 2017 using standardized chemical analyses and harmonized compilation procedures, following FAO/INFOODS and LATINFOODS guidelines. Nutritional information is expressed per 100 g of edible portion and includes macronutrients, energy, and minerals.

For this study, the entire nutritional table was digitized, resulting in an initial matrix of 136 records. Subsequently, a process of data cleaning, validation, and duplicate removal was carried out, yielding a final database of 129 unique foods, corresponding to the number of records reported in the original publication. All subsequent statistical analyses were performed on this consolidated database.

### 2.2 Classification of foods

The foods were grouped according to the classification established by the Cuenca Food Composition Table. Initially, twelve food categories were identified: cereals and derivatives, roots and tubers, legumes and derivatives, nuts and seeds, vegetables and derivatives, fruits and derivatives, meats and derivatives, eggs and derivatives, fish and shellfish, milk and derivatives, herbs and spices, and miscellaneous.

For analytical purposes, only groups with a sufficient sample size for robust statistical comparisons were retained. Groups with fewer than three observations were kept for descriptive analyses but excluded from inferential tests due to the inability to adequately estimate within-group variability.

### 2.3 Nutritional variables evaluated

Ten nutritional variables of interest were analyzed, obtained directly from the original database: energy (kcal 100 g^−1^), water (g 100 g^−1^), total protein (g 100 g^−1^), total fat (g 100 g^−1^), total carbohydrates (g 100 g^−1^), calcium (mg 100 g^−1^), iron (mg 100 g^−1^), potassium (mg 100 g^−1^), sodium (mg 100 g^−1^), and zinc (mg 100 g^−1^). These variables were selected because they consistently represent the main energy and mineral components available throughout the entire database.

### 2.4 Original analytical procedures

The values included in the Food Composition Table were obtained using internationally recognized official methodologies. Water content was determined by gravimetric drying at 105 °C. Total protein was estimated using the Kjeldahl method with Jones conversion factors. Total fat was determined by continuous Soxhlet extraction with prior acid hydrolysis. Carbohydrates were calculated primarily by difference, while dietary fiber was quantified using the AOAC 985.29 enzymatic-gravimetric method (16). Mineral content was determined by atomic absorption spectrometry and official colorimetric methods, depending on the nutrient being evaluated.

### 2.5 Quality control and data validation

The original database was constructed from representative samples collected in markets and supermarkets in the city of Cuenca, following INFOODS methodological recommendations. For each food item, descriptive information, taxonomic identification, and sample traceability were recorded. Chemical analyses were performed in triplicate using official AOAC methods, and the results were reported as mean and standard deviation. Quality control and compilation procedures followed FAO/INFOODS guidelines for food composition databases.

### 2.6 Statistical analysis

Initially, descriptive statistics were calculated for all nutritional variables, including mean, standard deviation, coefficient of variation, minimum and maximum values.

Differences between food groups were assessed using one-way analysis of variance (ANOVA). Before performing the one-way ANOVA, the underlying assumptions were evaluated. Normality of residuals was assessed using the Shapiro–Wilk test, whereas homogeneity of variances among food groups was evaluated using Levene’s test. When these assumptions were satisfied (*p* > 0.05), ANOVA was performed as planned. When significant differences were detected (*p* < 0.05), means were compared using Tukey’s HSD test to identify homogeneous clusters. When the assumptions of normality or homogeneity of variance were not satisfied (Shapiro–Wilk or Levene *p* ≤ 0.05), Welch’s ANOVA was used instead of the standard F-test to account for unequal variances and unequal group sizes, and *post hoc* pairwise comparisons were performed using the Games-Howell test, which does not assume equal variances or equal sample sizes.

Missing nutritional values were present mainly for protein (65 of 129 foods) and fat (55 of 129 foods), reflecting incomplete records in the original source table, with fewer missing values for calcium, iron, potassium, sodium, and zinc (9 foods each) and minimal missingness for energy, water, and carbohydrates. For descriptive statistics, ANOVA/Welch ANOVA, and Pearson correlations, missing values were handled through pairwise available-case analysis (i.e., each calculation used all foods with non-missing data for the variable(s) involved). Because PCA and hierarchical cluster analysis require a complete data matrix, missing values for these two analyses were imputed using the variable mean prior to Z-score standardization; this is a conservative approach that shrinks imputed cases toward the sample average and is noted here as a limitation given the substantial missingness in protein and fat.

The magnitude of the observed differences was quantified using the eta square effect size (η^2^), classifying it as small (η^2^ < 0.06), moderate (0.06 ≤ η^2^ < 0.14) or large (η^2^ ≥ 0.14).

The associations between nutrients were assessed using Pearson correlation coefficients. The suitability of the dataset for principal component analysis was evaluated using the Kaiser–Meyer–Olkin (KMO) measure of sampling adequacy and Bartlett’s test of sphericity. Variables were standardized as Z-scores before PCA to eliminate differences in measurement scales. Subsequently, principal component analysis (PCA) was applied using matrices standardized by *Z*-scores to identify multivariate patterns of nutritional composition and reduce the dimensionality of the data.

Hierarchical cluster analysis was performed using standardized variables, Euclidean distance as the dissimilarity metric, and Ward’s minimum variance (Ward. D2) linkage method. Cluster solutions ranging from two to eight groups were evaluated using the average silhouette width. The final four-cluster solution was selected based on the silhouette criterion together with the biological interpretability of the resulting nutritional profiles.

Cluster stability was assessed using 100 bootstrap resamples and Jaccard similarity coefficients. The gap statistic was also examined as an additional diagnostic for the number of clusters.

All statistical analyses were performed using R version 4.3.2 (R Foundation for Statistical Computing, Vienna, Austria). The main analyses were conducted using the packages stats, factoextra, FactoMineR, corrplot, agricolae, and cluster. Statistical significance was established at *α* = 0.05.

## 3 Results

### 3.1 Structure of the food composition database

The final database consisted of 129 unique foods representative of the traditional Ecuadorian diet, distributed across ten food groups (Table 1). The group with the highest number of entries was cereals and cereal products, with 36 foods (27.9%), followed by fruits and fruit products, with 28 foods (21.7%), and vegetables and vegetable products, with 15 foods (11.6%). Together, these three categories represented 61.2% of all foods included in the study.

**Table 1:** Distribution of foods included in the Ecuadorian food composition database according to food group.

Food group	*n*	Percentage (%)
Cereals and derivatives	36	27.9
Fruits and derivatives	28	21.7
Vegetables and derivatives	15	11.6
Roots and tubers	14	10.9
Meat and meat products	11	8.5
Miscellaneous foods	9	7.0
Legumes and derivatives	7	5.4
Milk and dairy products	6	4.7
Fish and seafood	2	1.6
Eggs and egg products	1	0.8
Total	129	100.0

The final database consisted of 129 unique foods obtained from the food composition table of Cuenca, Ecuador. Food groups containing fewer than three observations (eggs and fish) were retained for descriptive analyzes but were not considered in inferential statistical comparisons.

An intermediate representation was observed in roots and tubers (14 foods; 10.9%), meats and meat products (11 foods; 8.5%), miscellaneous items (9 foods; 7.0%), legumes and legume products (7 foods; 5.4%), and milk and dairy products (6 foods; 4.7%). Conversely, fish and shellfish (2 foods; 1.6%) and eggs and egg products (1 food; 0.8%) were the least represented categories in the database.

The distribution of the records shows a predominance of plant-based foods, particularly cereals, fruits, vegetables, and roots or tubers, which together represented 72.1% of the foods analyzed. Due to their small sample size, the fish and egg groups were retained for descriptive analyses but excluded from inferential comparisons to avoid weak estimates of within-group variability and statistical significance.

### 3.2 Nutritional composition of food groups

The nutritional composition of the food groups showed marked heterogeneity in energy, macronutrient, and mineral content (Table 2). Foods classified as miscellaneous had the highest average energy density (332.2 ± 182.3 kcal 100 g^−1^), followed by cereals and cereal products (301.7 ± 96.7 kcal 100 g^−1^), fish and seafood (299.0 ± 116.0 kcal 100 g^−1^), and milk and dairy products (288.5 ± 42.5 kcal 100 g^−1^). In contrast, the lowest energy concentrations were observed in vegetables and derivatives (35.1 ± 47.1 kcal 100 g^−1^), fruits and derivatives (85.3 ± 81.4 kcal 100 g^−1^) and roots and tubers (89.4 ± 61.0 kcal 100 g^−1^).

**Table 2:** Nutritional composition of Ecuadorian food groups (mean ± SD).

Food group	Energy (kcal/100 g)	Water (g/100 g)	Protein (g/100 g)	Fat (g/100 g)	Carbohydrates (g/100 g)	Calcium (mg/100 g)	Iron (mg/100 g)	Zinc (mg/100 g)
Cereals and derivatives	301.7 ± 96.7	33.9 ± 22.1	6.93 ± 2.53	9.25 ± 4.94	50.18 ± 18.94	75.2 ± 214.0	2.85 ± 4.65	0.92 ± 0.54
Roots and tubers	89.4 ± 61.0	78.1 ± 13.1	—	4.11 ± 3.21	19.05 ± 10.01	5.8 ± 6.1	0.30 ± 0.22	0.19 ± 0.12
Legumes and derivatives	143.6 ± 85.1	60.4 ± 25.2	12.51 ± 11.08	3.25 ± 3.05	18.69 ± 11.59	80.9 ± 99.7	4.50 ± 7.25	1.58 ± 1.72
Vegetables and derivatives	35.1 ± 47.1	90.8 ± 11.3	—	4.32	8.12 ± 9.65	26.3 ± 24.7	2.01 ± 5.54	0.26 ± 0.26
Fruits and derivatives	85.3 ± 81.4	78.6 ± 18.1	0.76	3.85 ± 2.25	19.54 ± 16.55	3.1 ± 3.7	0.26 ± 0.20	0.14 ± 0.10
Meat and meat products	236.7 ± 78.4	53.4 ± 10.7	21.85 ± 7.35	12.78 ± 9.38	8.55 ± 6.44	21.0 ± 21.8	1.85 ± 1.60	2.05 ± 1.39
Eggs and egg products	226.0	43.2	—	—	56.48	—	—	—
Fish and seafood	299.0 ± 116.0	45.3 ± 20.9	26.60 ± 4.10	18.30 ± 7.64	6.90 ± 7.78	17.0 ± 14.1	1.46 ± 0.94	0.71 ± 0.06
Milk and dairy products	288.5 ± 42.5	53.3 ± 3.4	5.00 ± 4.65	23.05 ± 5.35	18.29 ± 3.72	267.2 ± 193.7	0.38 ± 0.22	1.69 ± 1.09
Miscellaneous foods	332.2 ± 182.3	22.3 ± 28.3	4.45 ± 3.75	18.55 ± 8.39	52.30 ± 28.17	29.1 ± 61.3	0.98 ± 0.74	0.56 ± 0.60

Values are expressed as mean ± standard deviation per 100 g of edible portion. Groups with insufficient observations for a given nutrient are indicated by (−). Eggs (*n* = 1) and fish and seafood (*n* = 2) were retained for descriptive purposes but were excluded from inferential statistical analyses.

Water content showed a trend opposite to energy density. The highest concentrations were recorded in vegetables and derivatives (90.8 ± 11.3 g 100 g^−1^), fruits and derivatives (78.6 ± 18.1 g 100 g^−1^) and roots and tubers (78.1 ± 13.1 g 100 g^−1^), while the lowest values corresponded to miscellaneous (22.3 ± 28.3 g 100 g^−1^) and cereals and derivatives (33.9 ± 22.1 g 100 g^−1^).

Foods of animal origin had the highest protein content. The highest values were observed in fish and shellfish (26.6 ± 4.1 g 100 g^−1^) and meat and meat products (21.9 ± 7.3 g 100 g^−1^), followed by legumes and legume products (12.5 ± 11.1 g 100 g^−1^). Conversely, the lowest concentrations corresponded to fruits and fruit products (0.76 g 100 g^−1^), miscellaneous foods (4.45 ± 3.75 g 100 g^−1^), and milk and dairy products (5.00 ± 4.65 g 100 g^−1^).

Regarding lipid content, milk and dairy products had the highest average value (23.05 ± 5.35 g 100 g^−1^), followed by fish and shellfish (18.3 ± 7.6 g 100 g^−1^), miscellaneous (18.55 ± 8.39 g 100 g^−1^), and meat and meat products (12.78 ± 9.38 g 100 g^−1^). In contrast, legumes and legume products (3.25 ± 3.05 g 100 g^−1^), fruits and fruit products (3.85 ± 2.25 g 100 g^−1^), and roots and tubers (4.11 ± 3.21 g 100 g^−1^) had lower fat concentrations.

Carbohydrates were particularly abundant in miscellaneous (52.30 ± 28.17 g 100 g^−1^) and cereals and derivatives (50.18 ± 18.94 g 100 g^−1^), followed by fruits and derivatives (19.54 ± 16.55 g 100 g^−1^), legumes and derivatives (18.69 ± 11.59 g 100 g^−1^) and milk and derivatives (18.29 ± 3.72 g 100 g^−1^). Conversely, vegetables and derivatives (8.12 ± 9.65 g 100 g^−1^), meats and derivatives (8.55 ± 6.44 g 100 g^−1^) and fish and seafood (6.90 ± 7.78 g 100 g^−1^) showed the lowest contents.

Regarding minerals, milk and dairy products showed the highest average concentration of calcium (267.2 ± 193.7 mg 100 g^−1^), followed by legumes and legume products (80.9 ± 99.7 mg 100 g^−1^) and cereals and cereal products (75.2 ± 214.0 mg 100 g^−1^). The highest iron contents were recorded in legumes and legume products (4.50 ± 7.25 mg 100 g^−1^), cereals and cereal products (2.85 ± 4.65 mg 100 g^−1^), and vegetables and vegetable products (2.01 ± 5.54 mg 100 g^−1^). Meanwhile, the highest concentrations of zinc were observed in meats and derivatives (2.05 ± 1.39 mg 100 g^−1^), milk and derivatives (1.69 ± 1.09 mg 100 g^−1^) and legumes and derivatives (1.58 ± 1.72 mg 100 g^−1^).

The results show the coexistence of food groups with clearly differentiated nutritional profiles. While cereals and miscellaneous foods were characterized by high energy density and high carbohydrate content, vegetables, fruits, and tubers stood out for their high water content and lower caloric value. At the same time, meats, fish, milk, and legumes contained the highest levels of protein and essential minerals, reflecting their importance as sources of structural nutrients in the traditional Ecuadorian diet.

### 3.3 Statistical differences between food groups

Analysis of variance revealed significant differences between food groups for most of the nutritional variables assessed (Table 3). Energy showed high variability attributable to food group (*F* = 23.71; *p* < 0.0001), with a very large effect size (η^2^ = 0.614), indicating that 61.4% of the observed variability in energy content was associated with food group classification.

**Table 3:** One-way ANOVA and effect sizes for nutritional variables across food groups.

Variable	*F*	*p*-value	η^2^	ω^2^
Energy	23.71	<0.0001	0.614	0.587
Protein	20.38	<0.0001	0.641	0.606
Fat	8.97	<0.0001	0.491	0.433
Carbohydrates	18.61	<0.0001	0.560	0.528
Calcium	3.00	0.0043	0.178	0.129
Iron	1.70	0.106	0.113	0.056
Potassium	2.02	0.051	0.132	0.076
Sodium	1.79	0.086	0.118	0.061
Zinc	11.15	<0.0001	0.446	0.399

F, Fisher’s statistics; η^2^, proportion of the total variance in nutrient composition explained by food group classification; ω^2^, less biased estimate of the population variance attributable to food group classification. Higher η ^2^ and ω ^2^ values indicate that differences among food groups account for a larger proportion of the observed nutritional variability. According to Cohen’s criteria, η ^2^ values of 0.01, 0.06 and 0.14 represent small, medium and large effects, respectively.

A similar pattern was observed for protein content (*F* = 20.38; *p* < 0.0001; η^2^ = 0.641), which showed the largest effect size among all the variables analyzed. This result indicates that the differences between groups explained approximately 64.1% of the protein variability recorded in the database. Similarly, fat (*F* = 8.97; *p* < 0.0001; η^2^ = 0.491) and carbohydrate (*F* = 18.61; *p* < 0.0001; η^2^ = 0.560) content showed highly significant differences and large effect sizes.

The results of the assumption checks and the corresponding procedure used for each variable are summarized in Table 4. Residual normality was rejected for energy, protein, calcium, and zinc (Shapiro–Wilk *p* ≤ 0.001) and was borderline for fat (*p* = 0.072); homogeneity of variance was rejected for energy, protein, carbohydrates, and zinc (Levene *p* ≤ 0.05) but not for fat or calcium. Because at least one assumption was violated for energy, protein, carbohydrates, calcium, and zinc, Welch’s ANOVA and Games-Howell *post hoc* comparisons were used for these five variables instead of the standard F-test and Tukey’s HSD; fat was the only variable for which the standard ANOVA assumptions were fully met. Applying Welch’s correction did not change the substantive conclusions: energy [Welch *F*(7, 32.2) = 37.59, *p* ≤ 0.0001], protein (Welch *F*(4, 12.4) = 10.78, *p* ≤ 0.001; note that only five food groups had *n* ≥ 3 non-missing protein values), carbohydrates [Welch *F*(7, 33.5) = 20.06, *p* ≤ 0.0001], calcium [Welch *F*(7, 27.7) = 5.23, *p* ≤ 0.001], and zinc [Welch *F*(7, 28.2) = 13.95, *p* ≤ 0.0001] all remained highly significant, and Games-Howell comparisons identified the same broad pattern of pairwise differences (e.g., cereals and miscellaneous foods differing from vegetables, fruits, and roots/tubers in energy and carbohydrates; meats differing from plant-based groups in protein and zinc) described in the following paragraphs.

**Table 4:** Assumption checks and procedure used for each nutritional variable.

Variable	Shapiro–Wilk *p*	Levene *p*	Procedure used	Test statistic, df, *p*
Energy	<0.001	0.002	Welch ANOVA + Games-Howell	Welch *F*(7, 32.2) = 37.59, *p* ≤ 0.0001
Protein	<0.001	0.031	Welch ANOVA + Games-Howell	Welch *F*(4, 12.4) = 10.78, *p* ≤ 0.001
Fat	0.072	0.140	Standard ANOVA + Tukey HSD (assumptions met)	*F* = 8.97, *p* ≤ 0.0001 (as in Table 3)
Carbohydrates	0.291	0.003	Welch ANOVA + Games-Howell	Welch *F*(7, 33.5) = 20.06, *p* ≤ 0.0001
Calcium	<0.001	0.255	Welch ANOVA + Games-Howell	Welch *F*(7, 27.7) = 5.23, *p* ≤ 0.001
Zinc	<0.001	<0.001	Welch ANOVA + Games-Howell	Welch *F*(7, 28.2) = 13.95, *p* ≤ 0.0001

Among the minerals, calcium showed significant differences between food groups (*F* = 3.00; *p* = 0.0043; η^2^ = 0.178), while zinc showed a particularly marked effect (*F* = 11.15; *p* < 0.0001; η^2^ = 0.446). In contrast, iron (*p* = 0.106), potassium (*p* = 0.051), and sodium (*p* = 0.086) did not show statistically significant differences at the established significance level (*α* = 0.05).

These results demonstrate that the classification of foods into groups is a determining factor in explaining the variability observed in energy, macronutrients and some essential minerals, particularly protein, carbohydrates and zinc.

### 3.4 Nutrient correlation structure

**Figure 1:**
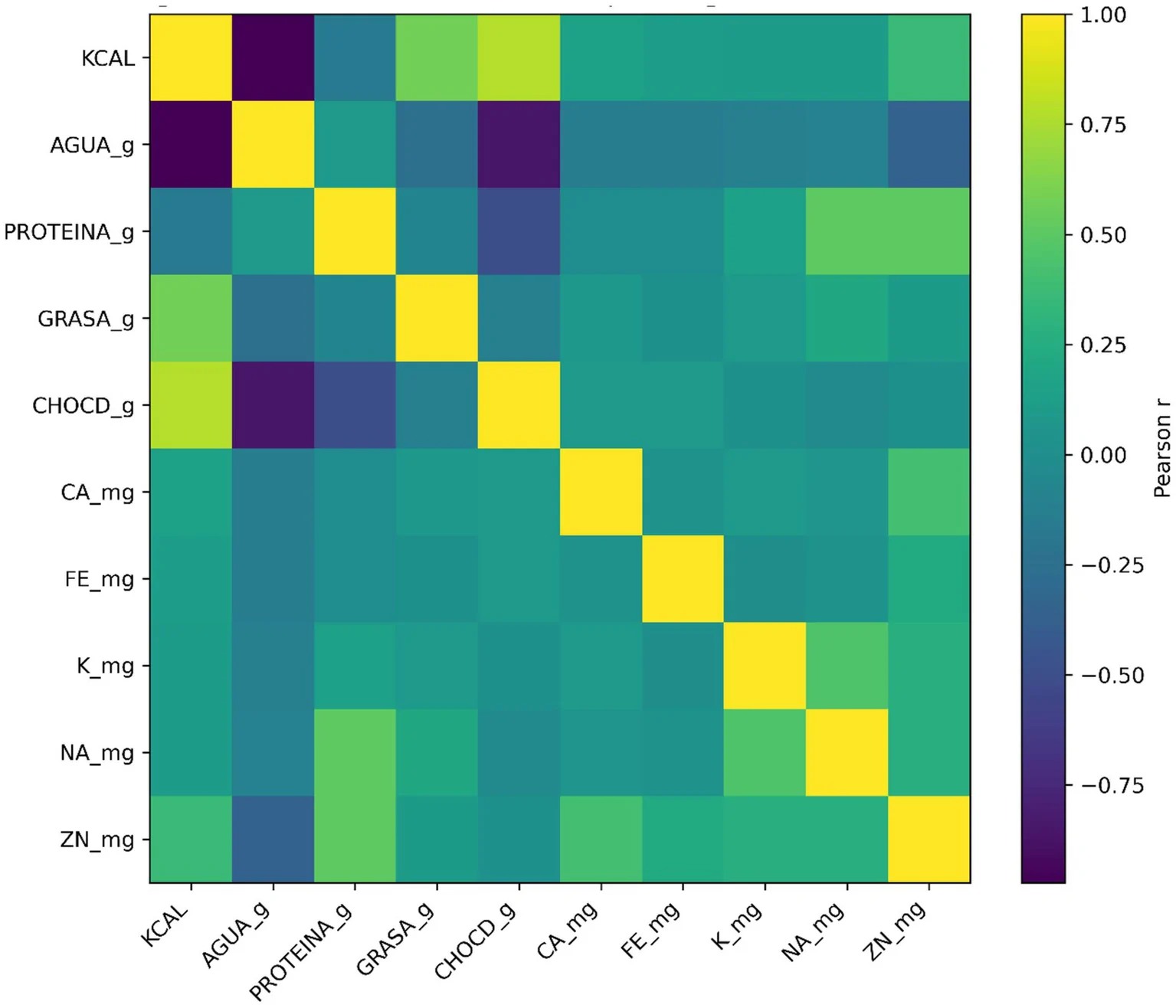
Pearson correlation heatmap among nutritional variables of the Ecuadorian food composition database. The heatmap displays Pearson correlation coefficients (*r*) among the nutritional variables included in the database. Positive values indicate direct associations, whereas negative values indicate inverse relationships. Color intensity is proportional to the strength of the correlation coefficient. The analysis included energy (KCAL), water (WATER), protein (PROTEIN), fat (FAT), carbohydrates (CHOCD), calcium (CA), iron (FE), potassium (K), sodium (NA), and zinc (ZN). Strong positive correlations indicate nutrients that tend to occur simultaneously within foods, whereas strong negative correlations reveal contrasting nutritional patterns among food groups.

The Pearson correlation matrix revealed well-defined associations between the main nutritional components of the evaluated foods (Figure 1). The strongest relationship observed was the negative correlation between energy and water content (*r* = −0.972), indicating that foods with higher moisture content consistently had lower energy density. This result reflects the diluting role of water on the concentration of energy nutrients and constitutes the main nutritional gradient present in the database.

Additionally, energy content showed a strong positive correlation with carbohydrates (*r* = 0.783), indicating that this macronutrient was one of the main determinants of the energy density of the analyzed foods. Furthermore, energy showed a moderate positive correlation with total fat (*r* = 0.579), confirming the combined contribution of both components to caloric intake.

Water content also showed a significant negative association with carbohydrates (*r* = −0.856), indicating that foods with high moisture content tended to have lower concentrations of available carbohydrates. This relationship was consistent with the composition observed in fruits, vegetables, and tubers, which are characterized by high water content and lower energy concentrations.

Proteins showed moderate negative correlations with carbohydrates (*r* = −0.498), suggesting a distinction between predominantly energy-rich foods and foods with structural or protein functions. Additionally, proteins showed moderate positive associations with sodium (*r* = 0.514) and zinc (*r* = 0.510), reflecting the contribution of animal-based foods and some legumes to the simultaneous provision of these nutrients.

Among the minerals, a moderate positive correlation was observed between calcium and zinc (*r* = 0.407), as well as between potassium and sodium (*r* = 0.457). Zinc also showed positive associations with energy (*r* = 0.364), protein (*r* = 0.510), and calcium (*r* = 0.407), suggesting that this micronutrient is primarily linked to more nutritionally dense foods.

The correlation structure reveals that the nutritional variability of Ecuadorian foods is primarily organized around two patterns. The first corresponds to an energy density gradient defined by the relationships between energy, water, and carbohydrates. The second is associated with a protein-mineral gradient characterized by positive associations between protein, zinc, sodium, and, to a lesser extent, potassium. These patterns suggest the existence of complex nutritional structures that justify the application of multivariate techniques to synthesize the information contained in the database.

### 3.5 Principal component analysis

The dataset was suitable for PCA according to the Kaiser–Meyer–Olkin measure and Bartlett’s test of sphericity (overall KMO = 0.537, indicating a mediocre but acceptable sampling adequacy per Kaiser’s criterion; Bartlett’s *χ*^2^(45) = 874.61, *p* ≤ 0.001). Individual measures of sampling adequacy (MSA) ranged from 0.172 for fat to 0.638 for sodium; fat’s low MSA reflects its substantial proportion of missing values and indicates that its individual contribution to the correlation structure should be interpreted with some caution, even though the overall KMO supported proceeding with PCA. To synthesize the complex nutritional structure observed in the database, a principal component analysis (PCA) was applied using previously standardized variables. The first two components together explained 49.6% of the total variability, with 30.1% corresponding to the first principal component (PC1) and 19.5% to the second principal component (PC2). Furthermore, the first five components accounted for more than 77% of the total variation, indicating adequate dimensionality reduction of the dataset.

The factor loadings of the first two components are presented in Table 5. PC1 was primarily associated with water content (0.552), energy (−0.552), and carbohydrates (−0.465). The magnitude and opposite direction of the energy and water loadings indicate that this component represents an energy density gradient, where foods with negative scores have higher concentrations of energy and carbohydrates, while those with positive scores are characterized by high water content and lower caloric value.

**Table 5:** Principal component loadings for the nutritional variables included in the Ecuadorian food composition database.

Variable	PC1	PC2
Energy (kcal)	−0.552	0.087
Water (g)	0.552	−0.061
Protein (g)	−0.214	0.482
Fat (g)	−0.328	0.271
Carbohydrates (g)	−0.465	−0.146
Calcium (mg)	−0.183	0.318
Iron (mg)	−0.102	0.194
Potassium (mg)	0.118	0.389
Sodium (mg)	−0.094	0.440
Zinc (mg)	−0.152	0.476

Loadings represent the contribution of each nutritional variable to the corresponding principal component. Positive and negative values indicate the direction of association with each component. Only the first two principal components are shown because together they explained 49.6% of the total variance observed in the dataset.

The eigenvalues and variance explained by all ten components were: PC1 = 3.03 (30.1%), PC2 = 1.96 (19.5%, cumulative 49.6%), PC3 = 1.16 (11.5%, cumulative 61.1%), PC4 = 1.06 (10.6%, cumulative 71.7%), PC5 = 0.97 (9.6%, cumulative 81.3%), PC6 = 0.94 (9.3%, cumulative 90.6%), PC7 = 0.54 (5.3%), PC8 = 0.34 (3.3%), PC9 = 0.06 (0.6%), and PC10 = 0.01 (0.1%). Following the Kaiser criterion (eigenvalue > 1), PC1 through PC4 would technically be retained, and the scree plot shows a first, sharper inflection between PC2 and PC3 followed by a more gradual decline through PC6, rather than a single unambiguous elbow. The interpretation focuses on PC1 and PC2 because these components capture the two dominant and readily interpretable nutritional gradients, namely the energy–water and protein–mineral gradients. In comparison, PC3–PC5, which explained 11.5, 10.6, and 9.6% of the variance, respectively, exhibited more diffuse loading patterns across variables, with no single nutrient loading above |0.45|, and represented secondary dimensions of nutritional variability. This interpretive approach emphasizes the components that provide the clearest biological and nutritional differentiation among the analyzed foods.

Meanwhile, PC2 was dominated by the variables protein (0.482), zinc (0.476), sodium (0.440), and potassium (0.389), forming a protein-mineral gradient. This pattern suggests that foods with high scores in this component simultaneously contain higher amounts of protein and essential minerals, particularly zinc and potassium.

**Figure 2:**
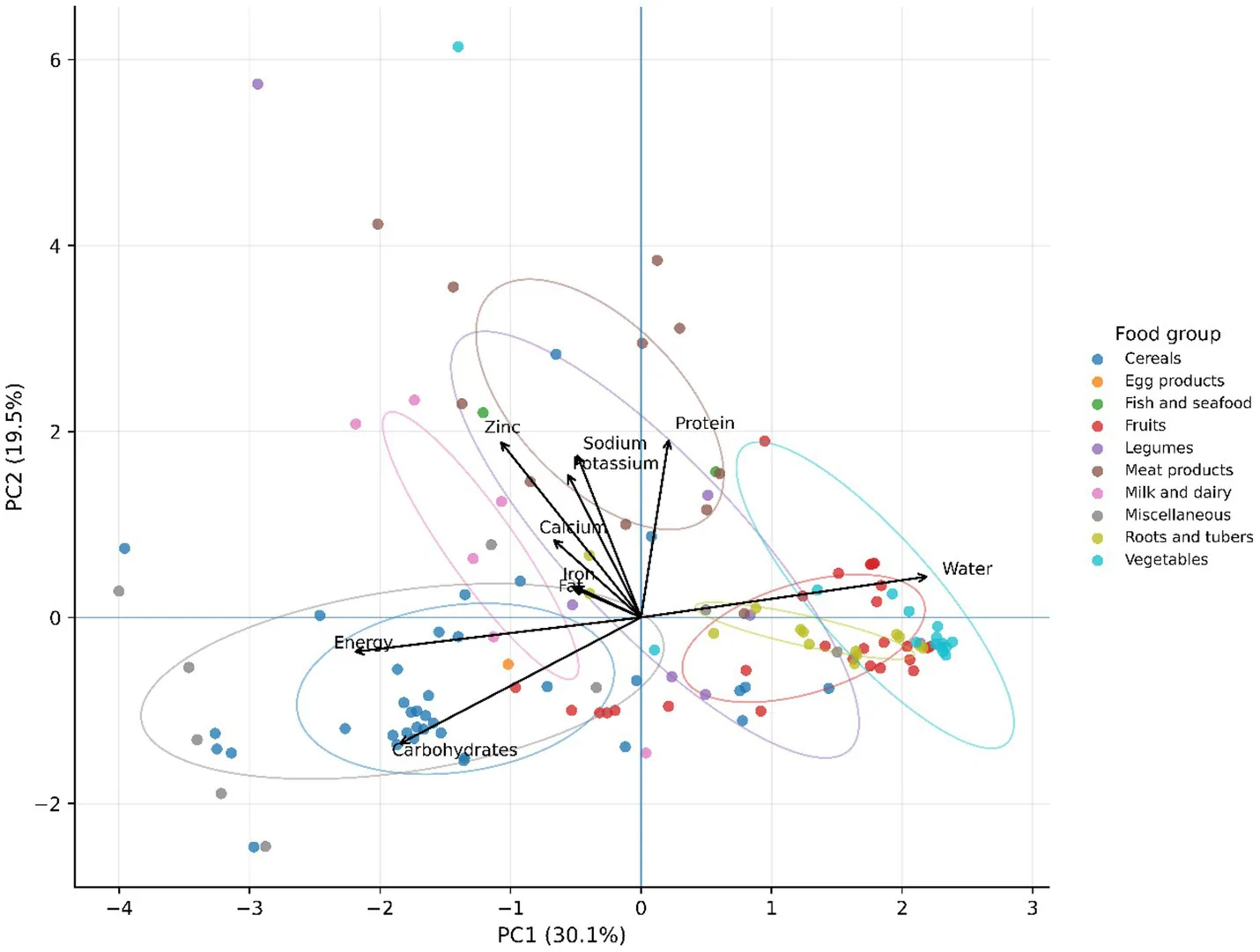
Principal component analysis (PCA) biplot showing the distribution of Ecuadorian foods according to their nutritional composition. The biplot represents the projection of food items onto the first two principal components (PC1 and PC2), which together explained 49.6% of the total variance. Arrows indicate the direction and relative contribution of nutritional variables. Foods located near a vector are positively associated with that nutrient, whereas foods located in the opposite direction show lower concentrations. PC1 primarily represents an energy-water gradient, while PC2 represents a protein-mineral gradient.

The distribution of foods within the space defined by the first two components revealed a clear differentiation in nutritional profiles (Figure 2). Cereals and various miscellaneous products tended to be located in the negative region of PC1, associated with higher energy and carbohydrate content. In contrast, fruits, vegetables, and tubers were predominantly concentrated in the positive region of the component, reflecting their high water content and lower energy density.

Foods of animal origin, particularly meats, fish, and some dairy products, were distributed primarily toward positive PC2 values, consistent with their higher protein and mineral concentrations. Similarly, certain legumes occupied intermediate positions between the two gradients, demonstrating a more balanced nutritional composition. Overall, the PCA confirmed that the nutritional variability of traditional Ecuadorian foods is fundamentally organized around two main axes: an energy density gradient and a protein-mineral gradient.

### 3.6 Nutritional grouping of foods

**Figure 3:**
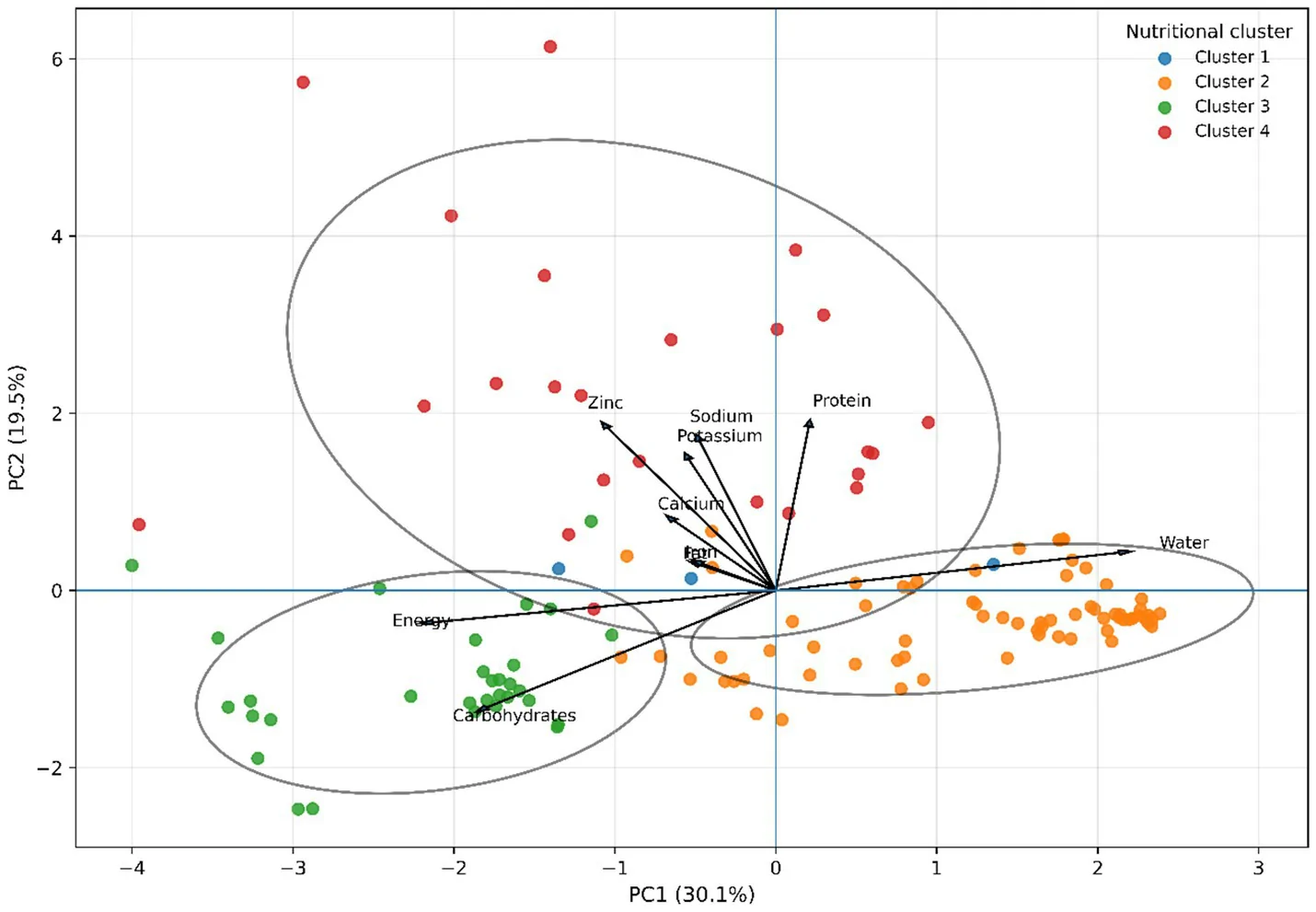
Principal component representation of the four nutritional clusters identified by hierarchical cluster analysis. Food items were assigned to four clusters using Ward’s hierarchical clustering method applied to standardized nutritional variables. Colors and ellipses represent nutritional clusters, whereas arrows indicate the direction and relative contribution of the nutritional variables to the multivariate structure. *PC1* and *PC2* explained 30.1 and 19.5% of the total variance, respectively. Clusters located in similar regions of the ordination space exhibit comparable nutritional profiles.

To identify shared nutritional patterns among the evaluated foods, hierarchical cluster analysis was performed using standardized nutritional variables, Euclidean distance, and Ward’s minimum-variance linkage method. Cluster validation was assessed for solutions ranging from two to eight groups. The four-cluster solution yielded an average silhouette width of 0.363 (silhouette widths for *k* = 2 to 8 ranged from 0.30 to 0.39, with a modest, gradual increase across this range rather than a single sharp peak at *k* = 4), while the gap statistic indicated no single unambiguous optimum: gap values increased monotonically from *k* = 1 through *k* = 7 without a clear local maximum, so the standard gap-statistic decision rule did not by itself identify k = 4 as optimal. Considering the statistical validation results together with the nutritional interpretability of the groups, the four-cluster solution was retained. This solution identified four compositional profiles (Table 6), whose distribution in the multivariate space defined by the first two principal components is shown in Figure 3.

**Table 6:** Nutritional characteristics of the food clusters identified by hierarchical cluster analysis.

Cluster	*n*	Energy (kcal/100 g)	Water (g/100 g)	Protein (g/100 g)	Fat (g/100 g)	Carbohydrates (g/100 g)	Calcium (mg/100 g)	Iron (mg/100 g)	Zinc (mg/100 g)
Cluster 1	3	146.0	56.7	10.3	13.0	15.4	84.2	23.37	1.42
Cluster 2	71	94.0	77.5	3.6	3.4	17.8	18.6	0.89	0.32
Cluster 3	31	366.9	19.8	7.2	11.4	61.2	42.3	1.71	0.78
Cluster 4	24	245.7	50.2	17.1	14.8	11.5	172.3	1.98	2.00

Cluster profiles were obtained from standardized nutritional variables using Euclidean distance and Ward’s minimum-variance linkage method (Ward. D2). The four-cluster solution was selected based on the average silhouette width, the gap statistic, and nutritional interpretability. Values correspond to cluster means.

Cluster 1 consisted of only three foods and was characterized by having the highest average iron content (23.37 mg 100 g^−1^), accompanied by intermediate values for energy (146.0 kcal 100 g^−1^) and fat (13.0 g 100 g^−1^). This group represented foods with exceptionally high mineral profiles, responsible for much of the variability observed for iron in the database.

Because Cluster 1 contained only three foods, a bootstrap stability analysis based on 100 resamples and following Hennig’s clusterboot approach was performed for all four clusters, measuring the Jaccard similarity between each original cluster and its best-matching counterpart in each resampled solution. Mean Jaccard similarities were 0.31 for Cluster 1, 0.54 for Cluster 2, 0.50 for Cluster 3, and 0.40 for Cluster 4. Relative to the conventional reference value of Jaccard ≥ 0.6, Clusters 2 and 3 showed the highest levels of reproducibility, followed by Cluster 4 and Cluster 1. For Cluster 1, 33% of the resamples reproduced a matching cluster with Jaccard ≥ 0.5, a result consistent with its smaller number of constituent foods. Accordingly, the four-cluster solution provides a coherent and interpretable representation of the main compositional gradients, while Cluster 1 identifies a distinctive mineral-rich nutritional profile within the analyzed dataset.

Figure 3 shows that the clusters were differentially distributed along the nutritional gradients defined by the principal components. Cluster 2 was located predominantly in the positive region of PC1, associated with the water vector and distant from the energy and carbohydrate vectors. This distribution coincides with the profile described in Table 6, characterized by high moisture content (77.5 g 100 g^−1^) and low energy density (94.0 kcal 100 g^−1^), typical features of fruits, vegetables, and some tubers.

Conversely, Cluster 3 was concentrated in the negative region of PC1, close to the energy and carbohydrate vectors. This pattern confirms that the foods belonging to this cluster have a high energy density (366.9 kcal/100 g) and a high carbohydrate content (61.2 g/100 g), constituting the main energy profile identified in the database. The proximity of this group to these vectors indicates that both variables were key in differentiating it from the other clusters.

Cluster 4 primarily occupied the positive region of PC2 and showed a spatial association with the protein, calcium, zinc, sodium, and potassium vectors. This distribution agrees with the mean values reported in Table 6, where this group presented the highest concentrations of protein (17.1 g 100 g^−1^), calcium (172.3 mg 100 g^−1^), and zinc (2.0 mg 100 g^−1^). Consequently, this cluster represents a nutritional profile dominated by foods of animal origin and dairy products with high protein-mineral density.

Cluster 1 consisted of only three foods and occupied an intermediate position in the multivariate space. Despite its small size, this group had the highest average iron content (23.37 mg 100 g^−1^), justifying its separation from the other clusters. The location of this group near the iron vector suggests that iron was the main factor responsible for its individualization within the overall nutritional structure.

The agreement observed between principal component analysis and cluster analysis demonstrates that the nutritional composition of Ecuadorian foods is primarily organized along two fundamental gradients: an energy density gradient associated with energy and carbohydrates, and a protein-mineral gradient associated with protein and micronutrients. These results show that integrating multivariate techniques effectively summarizes the nutritional complexity present in food composition databases and facilitates the identification of dietary profiles with clearly differentiated compositional characteristics.

### 3.7 Nutritionally outstanding foods and outliers

Descriptive and multivariate analyses identified foods with outstanding nutritional profiles within the database (Table 7). In terms of energy, the highest values corresponded to processed products and fried foods, notably fried salted plantain chips (505 kcal/100 g), fried sliced potatoes (494 kcal/100 g), fried yucca fries with salt (489 kcal/100 g), fried sweet plantain chips (482 kcal/100 g), and cream-filled cookies (467 kcal/100 g). These foods were primarily associated with the energy cluster identified in the cluster analysis and with the negative region of PC1 in the PCA.

**Table 7:** Foods with the highest nutritional values identified in the Ecuadorian food composition database.

Nutrient	Food	Value
Energy (kcal/100 g)	Salted fried plantain chips	505
Energy (kcal/100 g)	Fried potato chips	494
Energy (kcal/100 g)	Fried cassava chips with salt	489
Energy (kcal/100 g)	Sweet fried plantain chips	482
Energy (kcal/100 g)	Cream-filled cookies	467
Protein (g/100 g)	Raw dry lupin	34.06
Protein (g/100 g)	Fried pork	29.80
Protein (g/100 g)	Fried sea bass	29.50
Protein (g/100 g)	Grilled pork chop	29.20
Protein (g/100 g)	Grilled beef loin	28.60
Zinc (mg/100 g)	Raw dry lupin	5.22
Zinc (mg/100 g)	Selected meat products	>4.00
Calcium (mg/100 g)	Dairy products (highest) values)	Up to 607
Iron (mg/100 g)	Selected cereals and legumes	Up to 27.99

Values correspond to the maximum nutrient concentrations identified within the database. Foods were ranked according to their nutrient content per 100 g of edible portion.

Regarding protein content, the highest values were observed in raw dried lupin beans (34.06 g/100 g^−1^), fried pork (29.80 g/100 g^−1^), fried sea bass (29.50 g/100 g^−1^), roasted pork chop (29.20 g/100 g^−1^), and roasted beef tenderloin (28.60 g/100 g^−1^). These foods coincided with the positive regions of PC2 and with the cluster characterized by high concentrations of protein and minerals.

Regarding micronutrients, the highest calcium content was found in La Chonta cheese, fresh cheese, and other dairy products, while the highest iron concentrations were observed in certain cereals and legumes. Zinc levels were highest in raw dried lupin beans, meats, and some dairy products, confirming the close association between this mineral and high-protein food sources.

Outlier analysis identified several foods with exceptionally high nutritional compositions compared to the rest of the database. Among these, raw rye stood out for its calcium content, raw dried lupin beans for their protein and zinc concentrations, and certain dairy products for their high calcium levels. These findings were not considered analytical errors, but rather legitimate expressions of the nutritional diversity present in traditional Ecuadorian foods.

Taken together, the results show that the database contains foods with highly contrasting nutritional profiles, capable of significantly influencing the patterns observed through ANOVA, correlation analysis, PCA, and hierarchical clustering. Identifying these foods provides useful information for nutrition education programs, the design of dietary guidelines, and strategies for promoting traditional foods with high nutritional value.

## 4 Discussion

### 4.1 Nutritional heterogeneity of traditional Ecuadorian foods

The marked nutritional differentiation observed among food groups is a direct consequence of the biological and functional diversity that characterizes food systems. In this study, classification by groups explained between 44.6 and 64.1% of the variability observed for energy, protein, carbohydrates, and zinc, demonstrating that much of the nutritional composition is structured by the biological nature of foods. This behavior coincides with that reported by FAO/INFOODS, where the chemical composition of foods responds to specific physiological functions associated with energy storage, tissue formation, or metabolic regulation (17, 18).

The breadth of the observed nutritional profiles also reflects Ecuador’s high ecological and productive diversity. While cereals had average energy contents exceeding 300 kcal/100 g, fruits and vegetables registered values below 90 kcal/100 g. Similarly, dairy products reached average calcium concentrations above 260 mg/100 g, contrasting with values below 10 mg/100 g observed in several plant groups. These differences are consistent with the hypothesis proposed by Micoanski et al. (19). They pointed out that countries with high food biodiversity tend to have a wider range of nutritional profiles due to the coexistence of species with contrasting biological functions and chemical compositions.

The observed nutritional heterogeneity also has functional relevance from the perspective of human nutrition. No single food group simultaneously provides optimal amounts of energy, protein, and micronutrients, which is why dietary diversity is one of the fundamental principles of international nutritional recommendations (20). The coexistence of energy-rich foods, protein sources, and mineral-rich products promotes nutritional complementarity and contributes to improving nutrient adequacy at the population level (21). Consequently, the observed variability should not be interpreted as a limitation of the database, but rather as an inherent characteristic of diverse and potentially more resilient food systems.

Some of this variability can also be attributed to the different degrees of processing of the foods included in the database. Several studies have shown that refining, concentration, and industrial formulation processes substantially modify energy density and the content of critical nutrients, widening the nutritional gradients present in a population (22). This phenomenon is particularly relevant in Latin America, where traditional foods and industrialized products coexist within the same food systems (23).

The nutritional structure identified in Ecuadorian foods coincides with patterns previously described in food composition databases developed in Brazil, Colombia, Peru, and Mexico, where most of the nutritional variability is also concentrated between food groups rather than within them (24). This consistency suggests that the observed patterns follow general biological principles of nutrient organization and provides a solid basis for the application of multivariate tools designed to identify compositional gradients and complex nutritional profiles.

### 4.2 Energy density as the main nutritional gradient

Identifying energy density as the primary gradient of nutritional variation is consistent with one of the most widely described patterns in the literature on food composition. Several studies have demonstrated that energy is an integrating property of food matrices, summarizing the combined contribution of carbohydrates, fats, and water content within a single food (25). Consequently, when multiple food groups are analyzed simultaneously, energy density often emerges as one of the main factors responsible for nutritional differentiation among foods (26).

The strong opposition observed between energy and water content reflects a widely recognized physiological and physicochemical relationship. Water adds volume and weight to food without significantly contributing to its energy content; therefore, increases in moisture are usually associated with reductions in caloric density. This phenomenon has been documented in fruits, vegetables, and various fresh foods, where water contents exceeding 80% result in nutritional profiles characterized by low energy concentration per unit of weight (27). The association observed in Ecuadorian foods coincides with this general trend and suggests that moisture is one of the main structural determinants of nutritional composition.

The close association between energy and carbohydrates is also biologically expected. In plants, carbohydrates represent the main form of energy storage and constitute the metabolic basis of seeds, grains, and storage structures. For this reason, cereals and various derived products often occupy extreme positions within the energy gradients identified in food composition studies (28). Similar patterns have been reported in national databases developed in Latin America, Europe, and North America, where energy variability is strongly explained by differences in carbohydrate and fat content (29).

From a nutritional perspective, the dominance of this gradient has significant implications for diet quality. Energy density is one of the most widely used indicators for assessing the balance between caloric intake and nutrient concentration, as it directly influences total energy intake and the feeling of satiety (30). Diets characterized by a high proportion of water-rich foods tend to have lower energy densities and higher concentrations of fiber, vitamins, and bioactive compounds, while patterns dominated by high-energy foods can increase the risk of excessive calorie consumption when there is no adequate dietary balance (31).

The relevance of this gradient extends beyond the individual physiological realm and is related to contemporary changes observed in food systems. Several authors have pointed out that the nutritional transition experienced by numerous middle-income countries is characterized by an increasing availability of energy-dense foods, frequently associated with refining, concentration, or industrial formulation processes (32). The coexistence of traditional water-rich foods alongside energy-concentrated products observed in the Ecuadorian database precisely reflects this duality, where historical dietary patterns coexist with foods representative of increasingly industrialized food systems.

The emergence of energy density as the main axis of nutritional organization suggests that much of the compositional variability of Ecuadorian foods can be interpreted based on the balance between water, carbohydrates, and energy. This behavior coincides with observations made in multivariate studies of food composition, where energy content is often one of the factors with the greatest capacity to summarize the nutritional diversity present in large food databases (33).

### 4.3 Protein and mineral concentration in traditional Ecuadorian foods

The observed association between protein and minerals is one of the most consistent patterns reported in food composition studies. From a biological perspective, this relationship stems from the structural organization of animal and plant tissues. Proteins actively participate in the transport, storage, and utilization of numerous minerals, while elements such as zinc, iron, and calcium play essential roles in metabolic, enzymatic, and structural processes closely linked to protein metabolism (34). Consequently, foods with high protein density often also contain higher concentrations of certain micronutrients.

In foods of animal origin, this association is particularly evident due to the high metabolic activity of muscle tissue. Iron participates in proteins related to oxygen transport and storage, while zinc is involved in hundreds of protein-dependent enzyme systems (35). Similarly, dairy products are a significant source of calcium due to its biological role in the development and maintenance of mammalian bone tissue. These functional relationships explain why meat, fish, and dairy products are often grouped within nutritional profiles characterized by high concentrations of protein and minerals, a pattern frequently described in food databases from Europe, North America, and Latin America (36).

Legumes represent a particularly interesting case because they combine characteristics of both energy-rich and protein-rich foods. Unlike cereals, legume seeds store a considerable proportion of nitrogen as reserve proteins, significantly increasing their nutritional value. At the same time, they are one of the main plant-based sources of iron and zinc in many traditional food systems (37). This dual functionality explains why legumes often occupy an intermediate position between predominantly energy-rich foods and those characterized by high protein and mineral density.

From a public health perspective, the association between protein and micronutrients is particularly relevant because several of the minerals involved are among the nutrients whose intake is often insufficient in different regions of the world. Iron deficiency remains a leading cause of nutritional anemia, while inadequate zinc intake can affect growth, immune function, and various metabolic processes (37). In this context, the availability of foods that simultaneously provide high-quality protein and essential micronutrients represents a significant nutritional advantage for designing dietary strategies aimed at improving the nutritional adequacy of the population.

The emergence of a clearly defined protein-mineral axis in multivariate analyses suggests that the nutritional composition of Ecuadorian foods does not depend exclusively on energy gradients. On the contrary, a significant portion of the observed variability is associated with biological functions related to protein synthesis, mineral metabolism, and the storage of essential nutrients. This pattern aligns with results obtained in nutritional profiling studies based on principal component analysis, where protein, zinc, iron, and calcium typically form a set of closely related variables that allow for the differentiation of foods with high nutrient density from those primarily focused on energy intake (38).

### 4.4 Nutritional profiles revealed through multivariate analysis

The agreement observed between principal component analysis and hierarchical clustering suggests that the nutritional composition of Ecuadorian foods exhibits a consistent internal structure rather than a random distribution of nutrients. Although both methods employ different mathematical principles, they identified similar patterns primarily associated with energy density gradients and protein-mineral concentration. This methodological convergence is an indicator of analytical robustness, as it reduces the likelihood that the identified patterns are due to statistical artifacts specific to a particular technique (39).

The PCA allowed the reduction of the complexity of ten nutritional variables to two main axes capable of summarizing approximately half of the total variability in the database. This level of explanation is comparable to that reported in food composition studies conducted in Europe, Asia, and Latin America, where the first two components typically account for between 40 and 60% of the total variation due to the inherent complexity of food systems (40). The persistence of interpretable patterns despite this complexity indicates that a significant portion of the nutritional organization of foods can be described by a relatively small number of biologically significant gradients. Although the remaining principal components increased the cumulative explained variance to more than 77%, each individually accounted for a substantially smaller proportion of the total variability and mainly described more specific compositional differences, without revealing additional nutritional gradients comparable in biological relevance to those identified by PC1 and PC2.

The identification of four nutritional profiles through hierarchical grouping complements this interpretation by demonstrating that foods are not distributed continuously along these gradients, but rather tend to organize themselves into groups with relatively homogeneous compositional characteristics. This behavior aligns with the concept of a nutrient Profiling, according to which foods can be classified into distinct nutritional categories based on the simultaneous combination of multiple nutrients and not solely through individual indicators (41). From this perspective, the identified clusters represent functional nutritional profiles rather than simple statistical groupings.

The usefulness of these approaches is particularly relevant in food composition databases, where the simultaneous interpretation of numerous nutrients often hinders the identification of general patterns. Several authors have pointed out that multivariate methods allow large volumes of nutritional information to be transformed into more easily interpretable structures, facilitating comparisons between foods, the identification of dietary profiles, and the construction of nutritional classification systems. Consequently, the combination of correlation analysis, principal component analysis, and hierarchical clustering provides a more integrated view of nutritional composition than that obtained through conventional (42) univariate analyses.

The consistency observed between the gradients identified by the PCA and the profiles defined through clustering suggests that the nutritional diversity of Ecuadorian foods responds to shared biological and functional mechanisms. This finding reinforces the usefulness of multivariate approaches as tools for synthesizing the nutritional complexity of diverse food systems and provides a solid methodological basis for future applications related to food classification, nutritional quality assessment, and the design of evidence-based food strategies.

An important consideration is that the present analyses were based on the Food Composition Table of Cuenca (2018), whose analytical sampling was primarily conducted in the southern Andean region of Ecuador. Although the database includes numerous foods that are traditionally consumed across the country, it does not constitute a statistically representative sample of all Ecuadorian food diversity. Consequently, the nutritional patterns identified should be interpreted as representative of the analyzed database rather than the entirety of traditional Ecuadorian foods. Future updates incorporating foods from the Coast, Amazon, and Galápagos regions will allow broader national comparisons.

Furthermore, the present study was designed to generate new knowledge through the integrated statistical analysis of an existing analytical database rather than through the production of new compositional measurements. Thus, its principal contribution lies in identifying nutritional gradients, compositional relationships, and multivariate food profiles that were not explored in the original Food Composition Table, thereby complementing the nutritional information available from a new analytical perspective.

## 5 Conclusion

This research demonstrated that the nutritional composition of traditional Ecuadorian foods exhibits a highly organized structure, rather than a random distribution of nutrients. Classifying foods into groups explained a substantial proportion of the observed variability for energy, protein, carbohydrates, and zinc, with large effect sizes (η^2^ = 0.446–0.641), confirming that the biological and functional nature of foods is a fundamental determinant of their chemical composition. Correlation analysis identified a strong opposition between energy and water content (*r* = −0.972), as well as a close association between energy and carbohydrates (*r* = 0.783), indicating that energy density represents the main nutritional gradient present in the database. Simultaneously, the positive associations between protein, zinc, sodium, and potassium revealed the existence of a second gradient related to protein-mineral density. Multivariate analyses confirmed these patterns. The first two principal components explained 49.6% of the total variability and allowed for the identification of two fundamental axes of nutritional organization: an energy-water gradient and a protein-mineral gradient. Consistently, hierarchical cluster analysis identified four clearly differentiated nutritional profiles, characterized by foods with low energy density and high moisture content, energy-rich carbohydrate foods, foods with high protein-mineral density, and a small group of foods with exceptionally high iron concentrations. Traditional foods of high nutritional value were also identified, notably raw dried lupin beans as a significant source of protein and zinc, various dairy products as important sources of calcium, and several cereals and legumes with high iron content. Taken together, these results provide a comprehensive view of the nutritional diversity of the foods included in the Food Composition Table of Cuenca and demonstrate the usefulness of multivariate approaches for synthesizing complex food composition information. The findings provide a valuable basis for future research in nutrition and food composition science and may contribute to the progressive development of more comprehensive regional and national food composition databases. Beyond its scientific contribution, the multivariate framework proposed in this study may serve as a methodological basis for future developments in nutritional assessment and food classification once validated with broader and geographically representative food composition databases. Future studies should extend this approach by incorporating additional bioactive compounds and expanding food composition databases to include foods from other regions of Ecuador. Such developments will improve the geographical representativeness of future nutritional characterizations and facilitate broader applications of multivariate nutritional analyses at the national level.

## Data Availability

The original contributions presented in the study are included in the article/supplementary material, further inquiries can be directed to the corresponding author.
